# Lipid-Based Nanoparticle Functionalization with Coiled-Coil Peptides for *In Vitro* and *In Vivo* Drug Delivery

**DOI:** 10.1021/acs.accounts.3c00769

**Published:** 2024-03-26

**Authors:** Dennis Aschmann, Renzo A. Knol, Alexander Kros

**Affiliations:** Leiden Institute of Chemistry, Leiden University, Einsteinweg 55, 2333CC Leiden, The Netherlands

## Abstract

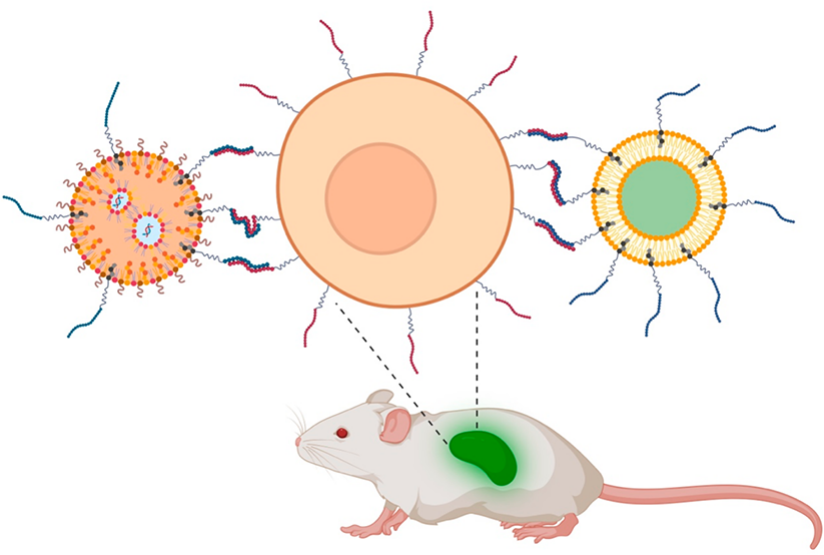

For the delivery of drugs, different nanosized drug carriers (e.g., liposomes, lipid nanoparticles, and micelles) have been developed in order to treat diseases that afflict society. Frequently, these vehicles are formed by the self-assembly of small molecules to encapsulate the therapeutic cargo of interest. Over decades, nanoparticles have been optimized to make them more efficient and specific to fulfill tailor-made tasks, such as specific cell targeting or enhanced cellular uptake. In recent years, lipid-based nanoparticles in particular have taken center stage; however, off-targeting side effects and poor endosomal escape remain major challenges since therapies require high efficacy and acceptable toxicity.

To overcome these issues, many different approaches have been explored to make drug delivery more specific, resulting in reduced side effects, to achieve an optimal therapeutic effect and a lower required dose. The fate of nanoparticles is largely dependent on size, shape, and surface charge. A common approach to designing drug carriers with targeting capability is surface modification. Different approaches to functionalize nanoparticles have been investigated since the attachment of targeting moieties plays a significant role in whether they can later interact with surface-exposed receptors of cells. To this end, various strategies have been used involving different classes of biomolecules, such as small molecules, nucleic acids, antibodies, aptamers, and peptides.

Peptides in particular are often used since there are many receptors overexpressed in different specific cell types. Furthermore, peptides can be produced and modified at a low cost, enabling high therapeutic screening. Cell-penetrating peptides (CPPs) and cell-targeting peptides (CTPs) are frequently used for this purpose. Less studied in this context are fusogenic coiled-coil peptides. Lipid-based nanoparticles functionalized with these peptides are able to avoid the endolysosomal pathway; instead such particles can be taken up by membrane fusion, resulting in increased delivery of payload. Furthermore, they can be used for targeting cells/organs but are not directed at surface-exposed receptors. Instead, they recognize complementary peptide sequences, facilitating their uptake into cells.

In this Account, we will discuss peptides as moieties for enhanced cytosolic delivery, targeted uptake, and how they can be attached to lipid-based nanoparticles to alter their properties. We will discuss the properties imparted to the particles by peptides, surface modification approaches, and recent examples showing the power of peptides for *in vitro* and *in vivo* drug delivery. The main focus will be on the functionalization of lipid-based nanoparticles by fusogenic coiled-coil peptides, highlighting the relevance of this concept for the development of future therapeutics.

## Key References

- YangJ.; BahremanA.; DaudeyG.; BussmannJ.; OlsthoornR. C. L.; KrosA.Drug Delivery via Cell Membrane Fusion Using Lipopeptide Modified Liposomes. ACS Cent Sci.2016, 2( (9), ), 621–63010.1021/acscentsci.6b0017227725960 PMC5043431.^1^*This report investigates a new method for direct drug delivery into the cytosol of live cells in vitro and in vivo. A pair of complementary coiled-coil lipopeptides were used to achieve targeted membrane fusion between liposomes and live cells*.
- YangJ.; ShimadaY.; OlsthoornR. C. L.; Snaar-JagalskaB. E.; SpainkH. P.; KrosA.Application of Coiled Coil Peptides in Liposomal Anticancer Drug Delivery Using a Zebrafish Xenograft Model. ACS Nano2016, 10( (8), ), 7428–743510.1021/acsnano.6b0141027504667 .^2^*Incorporating the fusogenic lipopeptide E4 into the bilayer of liposomes enables selective targeting of HeLa cells expressing the complementary fusogenic peptide K4 in vitro and in vivo*.
- ZengY.; ShenM.; SinghalA.; SevinkG. J. A.; CroneN.; BoyleA. L.; KrosA.Enhanced Liposomal Drug Delivery Via Membrane Fusion Triggered by Dimeric Coiled-Coil Peptides. Small2023, 19, e230113310.1002/smll.20230113337199140 .^3^*Comparison of different fusogenic lipopetides in order to optimize the delivery of drugs encapsulated in liposomes through cell membranes. In this particular case, the drug doxorubicin was used and the antitumor response was investigated*.
- ZengY.; Estapé SentiM.; LaboniaM. C. I.; PapadopoulouP.; BransM. A. D.; DokterI.; FensM. H.; van MilA.; SluijterJ. P. G.; SchiffelersR. M.; VaderP.; KrosA.Fusogenic Coiled-Coil Peptides Enhance Lipid Nanoparticle-Mediated mRNA Delivery upon Intramyocardial Administration. ACS Nano2023, 17( (23), ), 23466–2347710.1021/acsnano.3c0534137982378 PMC10722601.^4^*Building upon previous reports, we expanded our scope to lipid nanoparticles (LNPs) as delivery vehicles. Here we decorated the LNPs and cells with fusogenic peptides to overcome the endocytic pathway, leading to increased protein expression levels compared to those of plain LNPs.*

## 1 Introduction

The field of drug delivery has developed a broad range of vehicles for the administration of therapeutics. These vehicles are required to make poorly soluble drugs bioavailable, protect therapeutics against degradation, and prevent toxic compounds from interacting with the biological environment before they reach the desired target. Nowadays FDA-approved formulations such as Doxil, Onpattro, and Comirnaty based on nanoparticles have made their way to patients, where they contribute to human health care.^5^

A majority of nanoparticles reach their site of action through passive targeting, making them dependent on physiological and/or pathological conditions. Anyway, particles depending on passive targeting suffer from low drug concentrations in affected regions, leading to low therapeutic effects.^6^ Simultaneously, off-targeting leads to side effects which can be worse than the disease itself.^7^ To solve these problems, many groups around the globe are working on strategies to design nanoparticles with more precise targeting capability and increased endosomal escape.

The most common and promising approach is thereby the surface functionalization of nanoparticles to yield new properties such as improved circulation time, increased uptake, and/or targeted delivery. Depending on the choice of nanoparticle system, different approaches might be suitable to conjugate moieties fulfilling specific functions to the surface. Furthermore, the linker between the moiety and anchor plays a crucial role in the impact of function as well as on the stability of the resulting nanoparticles.

In order to alter the particle properties, a huge variety of small molecules or biomolecules can be used as moieties that specifically bind to surface-exposed receptors. Therefore, research groups are using different classes of biomolecules, such as small molecules, nucleic acids, antibodies, aptamers, and peptides.^8^ In particular, peptides are highly specific ligands for surface-exposed receptors. Additionally, peptides are easy as well as cheap to produce and are already known to improve uptake/targeting to desired regions or disease patterns.

However, there is no universal approach suitable for all possible combinations of peptides and nanoparticles. Therefore, this Account discusses how the surface of lipid-based nanoparticles can be functionalized and important matters in this context. Furthermore, cell-penetrating peptides (CPPs) are discussed and compared with fusogenic coiled-coil peptides for high delivery efficiency and targeting purposes.

## 2 Cell-Penetrating Peptides (CPPs)

In the past few decades, many peptides able to bind different cellular targets with high affinity were developed or discovered in order to fulfill specific functions. The most common examples are cell-penetrating peptides (CPPs), which are peptides efficiently crossing the plasma membrane, and cell-targeting peptides (CTPs) promoting selective delivery. In this Account, we focus on the cellular uptake through the plasma membrane of the cells, which should ultimately result in the release of a payload in the cytoplasm, so we focus here mainly on the CPPs.

The first known example of CPPs is the transactivating transcriptional activator (TAT) discovered in 1988 used by human immunodeficiency virus 1 (HIV-1). Nowadays, a huge variety of CPPs has been developed and divided into cationic, hydrophobic, and amphipathic CPPs.^9^ All of these CPPs are short amino acid oligomers (5–30 amino acids).^10^

Cationic peptides are highly charged and include derivatives of TAT, penetratin, and polyarginines. The positively charged amino acids (lysine and arginine) form hydrogen bonds with negatively charged phosphate, sulfate, and carboxylic groups of cell membrane constituents, leading to cellular internalization.^11^

Hydrophobic CPPs consist mainly of nonpolar residues, leading to less charged sequences compared to the other groups of CPPs. These lipophilic sequences show high affinities for hydrophobic domains exposed to cellular membranes, a requirement for cellular internalization. It is assumed that this family of peptides can be spontaneously internalized by an energy-driven pathway, which is different from other classes of CPPs.^12^

Amphipathic peptides contain polar (hydrophilic) and hydrophobic (lipophilic) regions. The polar regions are mainly represented by lysine and arginine, while the hydrophobic regions consist of alanine, leucine, isoleucine, and valine. If they are linked to a hydrophobic domain, then they can be chimeric, allowing the peptide to address cell membranes and nuclear location signals (NLS). Pep1 and MPG are examples of this peptide class and contain parts of HIV glycoprotein 41 or tryptophan-rich regions combined with the NLS of the large T antigen of simian virus 40 (SV40) (KKKRKV) (Table 1).^13−18^

**Table 1:**
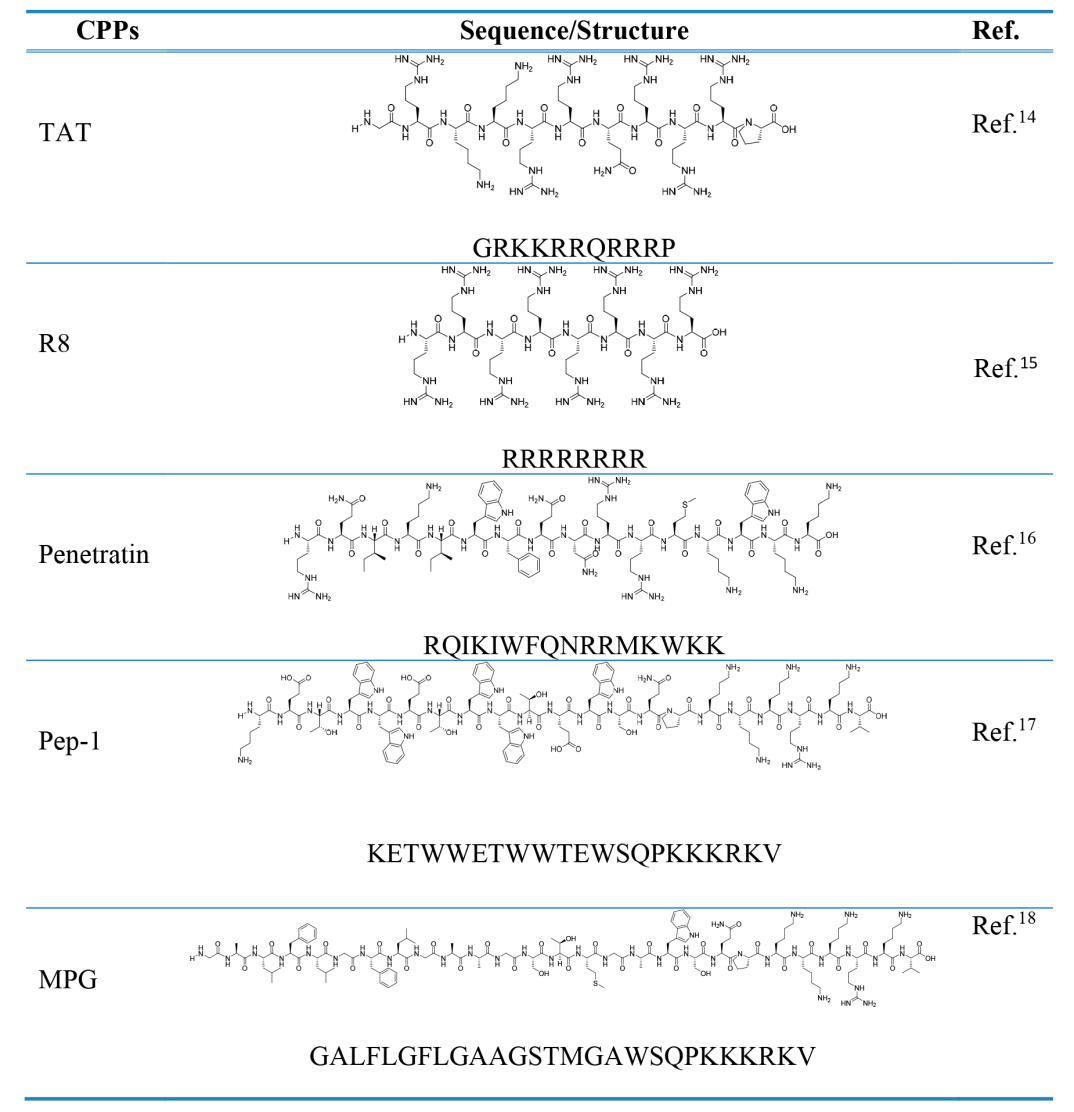
CPPs Described in This Account

What makes CPPs particularly attractive for pharmaceutical applications is their ability to penetrate some barriers, such as skin, the blood–brain barrier (BBB), and the cornea or conjunctiva of the eyes.^19,20^ This makes it possible to replace unpleasant, potentially dangerous, and painful injections with creams, drops, and soothing sprays.

Despite intensive research over the last three decades on effective CPPs, there is no CPP or composition containing CPPs that has been approved by the Food and Drug Administration (FDA). CPPs have a few drawbacks, making them unsuitable for drug applications. First, CPPs suffer from low cell and tissue selectivity as well as cytotoxicity, which is often observed. Additionally, most CPPs are dependent on an endocytic pathway for cell entry. This mechanism is often limited by insufficient escape from endosomes, degradation, restricted diffusion, and/or a lack of nuclear uptake.^21^ In terms of vaccination purposes, CPPs face costs that are too high and the necessity of transfecting immune cells, which are difficult to transfect.^22^

Furthermore, it is difficult to make predictions about the transport efficiency or cell selectivity, as these may depend mostly on organs or tissues as well as on the conjugated drug or nanoparticles.

Besides the described well-known CPPs, fusogenic coiled-coil peptides can be used to bind selectively with high affinity to a partner peptide, allowing them to overcome the plasma membrane of cells. This bypasses the endosomal pathway, making them attractive components for the preparation of vehicles for precise delivery with high transport efficiency.^23^

## 3 Fusogenic Peptides

Fusion processes such as membrane fusion are crucial for cellular survival. They facilitate the formation and downstream processing of transporter vesicles, intracellular endosomes, and extracellular synaptic vesicles.^24^ In nature, the fusion of membranes is mainly regulated by so-called SNARE (soluble *N*-ethylmaleimide-sensitive factor attachment protein receptor) proteins, a protein family identified by a well-conserved tetrameric coiled-coil (CC) motif with 60–70 amino acids per α-helix.^25^ SNARE proteins are responsible for a huge variety of tasks in the fusion machinery; they signal the position where fusion should occur, and they provide (part of) the necessary platform of interactions to overcome multiple energy barriers to fuse initially stable membranes.^24^

In the past few years, one of the most studied-in-detail SNARE-mediated membrane fusion processes has been the release of neurotransmitters. Neurotransmitters are enveloped by vesicles decorated with a single SNARE protein able to interact with two other SNARE proteins associated with the axonal presynaptic membrane to release neurotransmitters into the synaptic cleft.^26^ Inspired by the precise SNARE protein-based fusion machinery, numerous simplified model systems with a range of molecules have been developed over the past few years. Thereby, synthetic fusogens based on deoxyribonucleic acid (DNA), peptide nucleic acid (PNA), peptides, and other small-molecule recognition complexes have been studied.^27,28^

**Figure 1:**
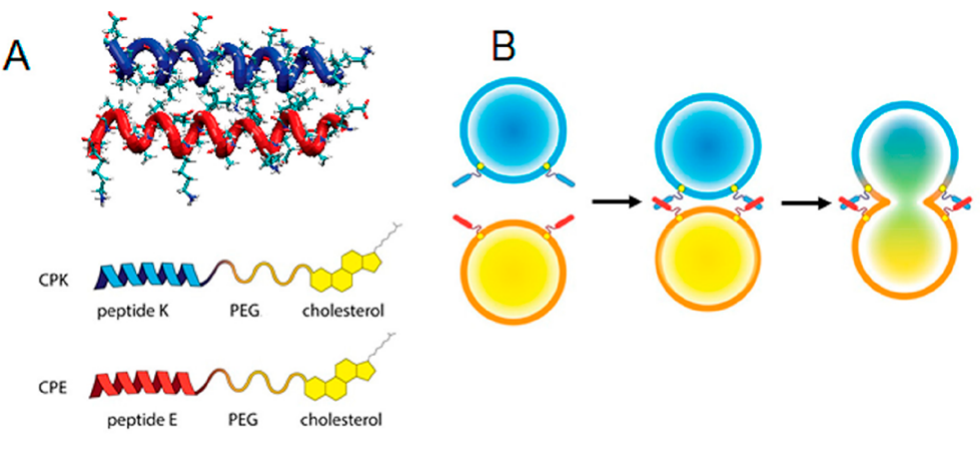
Schematic representation of (A) coiled-coil structure between peptides E and K (adapted from PDB 1UOI), (B) targeted liposome fusion mediated by coiled-coil formation between CPE4-modified liposomes and CPK4-modified liposomes. Reproduced with permission from ref (1). Copyright ACS 2016.

These simplified model systems are based on strong interactions between fusogens bound to the membrane and those on the surface of particles (e.g., liposomes). The interaction between fusogens brings the particles (of liposomes, vesicles, or LNPs) and membrane (of cells or liposomes) close together, ultimately leading to fusion. One successful example of peptide-based fusogens is the heterodimeric coiled-coil pair E3 and K3 (with the amino acid sequences (EIAALEK)_3_ and (KIAALKE)_3_ respectively). Adding a poly(ethylene glycol) (PEG)-based spacer and a lipid anchor enables immobilization into the membrane (Figure 1).

The PEG-spacer length, the peptide length, and the orientation all influence the fusogen’s performance. Studies showed that a construct of cholesterol, PEG4, and peptide (E4 or K4) efficiently promotes membrane fusion *in vitro* as well as *in vivo*. Furthermore, E4 and K4 take over different tasks, being crucial for efficient fusion; peptide K4 facilitates the formation of lipid protrusions by spontaneous membrane insertion, whereas peptide E4 acts as an orthogonal connection to allow the approximation of opposing lipid-based particles.

The lipopeptide-based fusion system CPE/CPK has already been used in many applications, such as cell sorting,^29^ liposome fusion,^25,26,30^ liposome cell fusion,^1,3,31^ lipid nanoparticle cell fusion,^32^ cell–cell fusion,^33^ and anion transporter delivery to membranes.^34^

## 4 Functionalization of Lipid-Based Nanoparticles

**Figure 2:**
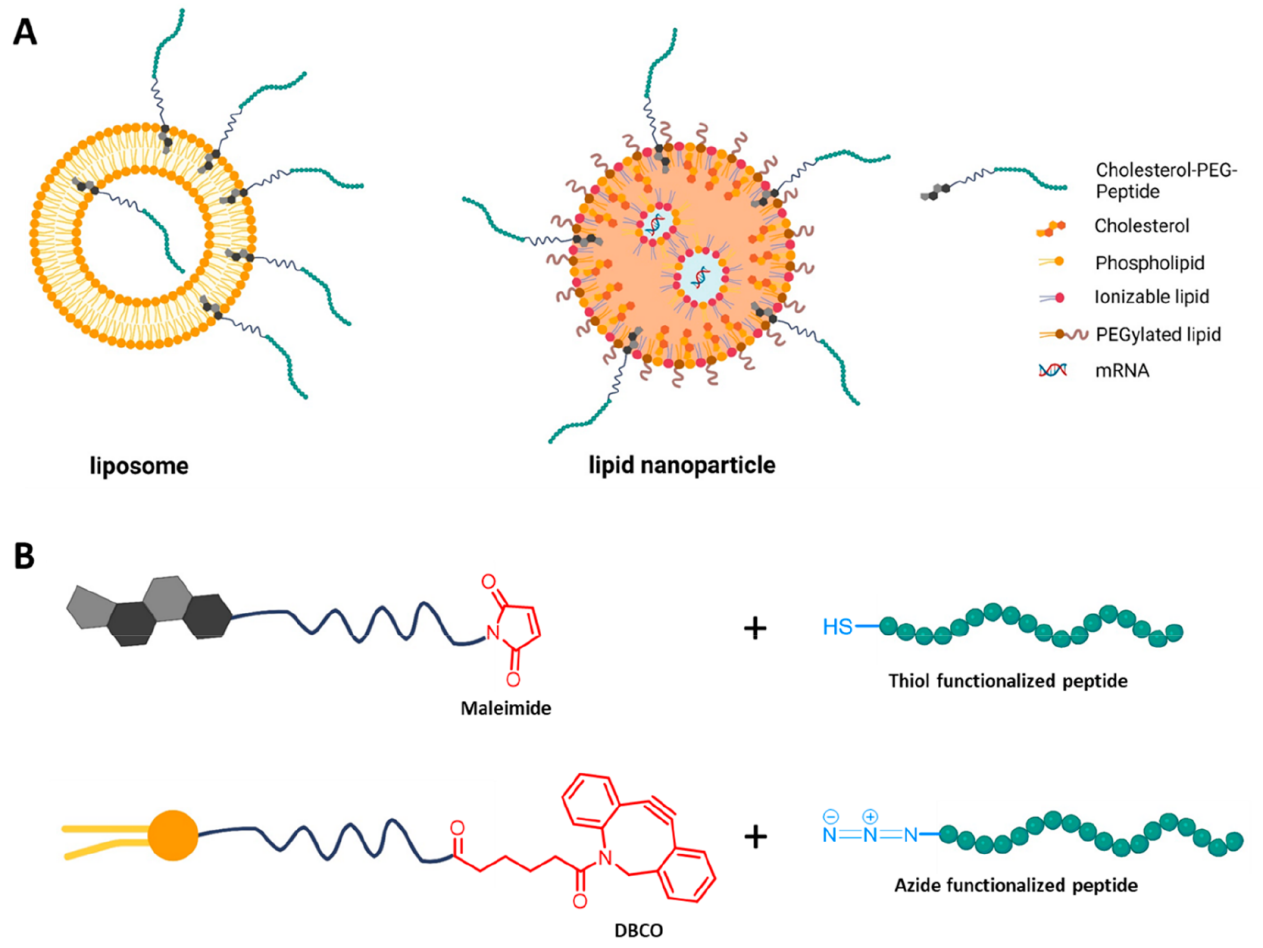
Surface functionalization of lipid-based nanoparticles. (A) Illustration of surface-functionalized liposomes and lipid nanoparticles. (B) Common postfunctionalization methods. Created with BioRender.com.

Liposomes and lipid nanoparticles rely on the self-assembly of single lipid molecules and are manufactured by different methods (e.g., extrusion, microfluidics, sonication, and electroformation). Liposomes can be produced by hydrating a lipid film followed by sonication or extrusion until the desired size and monodispersity are achieved and thereby encapsulate a drug of interest.^35^ On the other hand, lipid nanoparticles are mainly formed by rapid mixing of lipids dissolved in ethanol and mRNA dissolved in an acidic buffer (commonly citrate or acetate buffer, pH 4.0). By electrostatic interactions between mRNA/siRNA and cationic-charged lipids, the assembly of LNPs takes place, followed by hydrophobic and van der Waals interactions, leading to stable particles encapsulating the therapeutic.^36^ Since the production method of such assemblies differs, it is necessary to adapt the functionalization method. It is of utmost importance to consider that the interaction of the CTPs with a surface-expressed receptor must be accessible; therefore, the peptide must be exposed on the surface of the particles. In order to achieve this, the targeting moiety has to consist not only of the peptide itself but also a hydrophobic anchor (commonly cholesterol or a lipid) and a linker unit (often PEG) which connects the hydrophobic anchor with the targeting peptide (Figure 2A).

Hydrophobic anchors ensure that the compound embeds itself into the lipid membrane and remains there while the lipid-based nanoparticles circulate freely *in vivo*, and the bound peptide can interact with the receptor of interest, ensuring specific cell targeting as well as uptake. Cholesterol and the phospholipid 1,2-distearoyl-sn-glycero-3-phosphoethanolamine (DSPE) are frequently used for this purpose, as they are integrated particularly stably into the membrane of lipid-based particles.

Linkers fulfill the function of linking the hydrophobic anchor and the CTP to each other. Furthermore, the choice of length can reduce the interaction of the peptide with the lipid surface of the particles or increase the interaction with the receptor. It should be mentioned that the targeting efficiency decreases when the linker is too long and an additional stealth effect can occur: lipid-bound polyethylene glycol sterically hindering the nanoparticle surface as well as targeting moieties, improving the circulation properties but reducing the cell-specific uptake.

CPPs have the important function of interacting with the surface-exposed receptors of cells to enable cellular internalization in the first place.^37^ Furthermore, it is important to attach the linker containing the anchor without influencing the native interaction with the target receptor. Depending on the peptide, there may be solubility problems due to the polarity, which may be compensated for by the length and polarity of the linker. Nevertheless, it should be noted that very hydrophobic peptides adsorb on the surface of the particles and reduce the interaction with the aqueous medium. On the other hand, very strongly charged peptides can ensure that the particles no longer circulate freely and are immediately taken up at the injection site. The number of surface-exposed peptides certainly plays a decisive role, but not every peptide that exhibits unconjugated targeting must also do so in the bound state.

In liposomes, the hydrophobic anchor is inserted in the lipid bilayer upon extrusion/sonication since the lipids constantly rearrange until the desired particles are generated. Through this method, a population of the peptide will point into the aqueous core of the liposomes since the arrangement is random. To ensure that the peptide is exposed only on the exterior surface, copper-free click chemistry can be used to connect cholesterol and the linker with the CPP postformulating the particles (Figure 2B).

In contrast, LNPs are manufactured by microfluidic mixing, and once assembled, the size and polydispersity index (PDI) cannot be adjusted by extrusion. Therefore, care should be taken when choosing the anchor, linker (especially the length), and peptide to ensure that the particle formulation process is not influenced. Too many charged residues in the peptide sequence can lead to competitive interactions with mRNA disrupting the arrangement of the particle. Furthermore, if the whole construct is too hydrophobic, then the CPP inserts in the interior of the LNP or attaches to the surface, preventing interaction with cell surface-exposed receptors. To overcome this issue, postmodification by thiol-maleimide chemistry or copper-free click chemistry can be used to ensure that no adverse interactions occur.^38^

## 5 Lipid-Based Nanoparticles Functionalized with CPPs

Despite many years of research, the field of “nanomedicine” still has major challenges. Nanotherapeutics still suffer from off-targeting, insufficient endosomal escape efficiency, and hepatic and renal clearance. Furthermore, nanomedicine is not able to tackle major disease due to barriers preventing the accessibility of affected tissue/organs (e.g., BBB, skin, or the mucosal barrier).

However, liposomes and LNPs in particular have emerged as very successful and biocompatible delivery systems. PEGylation of such particles enabled long circulation times. The uptake and efficiency of the endosomal escape have already been improved and now enable pharmaceutical applications (e.g., Doxil, Onpattro, and Comirnaty). Nevertheless, the uptake of particles via the endosomal pathway is responsible for the fact that the efficacy of all formulations does not reach its full potential. Therefore, ways need to be found to overcome or bypass the endosomal pathway more efficiently. In addition, lipid-based particles cannot overcome physiological barriers without further functionalization.

To overcome physiological and cellular barriers and/or to achieve successful targeting, a huge variety of nanoparticles have been functionalized with CPPs and CTPs, including liposomes, lipid nanoparticles (LNPs), polymeric nanoparticles, gold/metal nanoparticles, and silica quantum dots.^39−50^

It is therefore of special interest to highlight these two vehicles in relation to surface functionalization.

### 5.1 Surface-Functionalized Liposomes

Since its FDA approval in 1995, Doxil has been one of the most successful liposomal formulations. Therefore, it has already been functionalized with peptides to reduce off-targeting and associated side effects.^51^ Studies showed that the surface of the Doxil formulation can be modified by copper-free click chemistry, leading to a functionalization of 1% of the total lipid amount with a peptide called p700. This approach allowed the altering of the properties yielding enhanced localization in tumor tissue.^52^ Furthermore, specific targeting of glioma can be achieved by inserting a tandem peptide consisting of R8 (CPP) and RGD (CTP). This peptide was conjugated via thiol maleimide chemistry to a DSPE-PEG2000 lipid-based spacer and inserted in the bilayer during the formulating process. R8-RGD increased the cellular uptake 2-fold and nearly 30-fold compared to separate R8 and RGD, respectively, and *in vivo* studies in mice showed that R8-RGD-liposomes could be efficiently delivered into the brain and selectively accumulated in the glioma foci.^53^ Further examples showed that the functionalization with a peptide called ApoEdp (CFGGGLRKLRKRLLLRKLRKRLL) yields 3.9-fold higher accumulation in the brain compared to the nonmodified liposome formulation.^47^

Uhl et al. demonstrated that the modification of liposome surfaces with a cell-penetrating peptide (cyclic R9 peptide) allows mucosal permeation. Therefore the cyclic R9 (cR9) peptide was conjugated to a PEG12 linker containing a maleimide group, which was bound to a thiol-modified phospholipid to achieve a construct of lipid-PEG12-cR9. The liposomal formulation functionalized with 3 mol % lipid-PEG12-cR9 was analyzed in Ussing chamber studies. Thereby a high mucosal uptake of the glycopeptide antibiotic vancomycin was shown. Afterward the efficacy was proofed *in vivo* in naive rats, where a highly increased oral bioavailability was obtained for vancomycin (a known drug to be minimally absorbed). In contrast, the administration of liposomes without CPP leads to a significantly lower bioavailability.^54^

A different example shows that CPP-modified liposomes are not always beneficial, depending on the encapsulated drug. In this case, liposomes encapsulated insulin and were functionalized with the peptide TAT or PNT to promote drug penetration through porcine nasal mucosa. However, the TAT/PNT liposomes promoted lower levels of release and permeation through the nasal mucosa of porcine compared to those of the liposomal system without functionalization. This finding is likely due to the electrostatic interaction between insulin and CPPs. The complexation between them reduces the incorporation of the drug by liposomes, inhibiting the peptide’s absorption-promoting activity.^55^

### 5.2 Surface-Functionalized LNPs

While liposomes are mainly used for the delivery of toxic drugs to treat cancer, LNPs are used to encapsulate mRNA or siRNA to express/silence proteins. The therapeutic potential of LNPs has a broad range including nonviral vaccines, protein replacement therapies, cancer immunotherapies, cellular reprogramming, and genome editing.^56^ Precise delivery of the therapeutic may also be advantageous and necessary in these cases. It is therefore not surprising that the approach of surface functionalization with peptides which already yielded promising results for liposomes has also been applied to LNPs.

In this regard, the well-known RGD peptide has already been used. Functionalized with lipid chains, it comprised up to 20% of the total lipid amount of LNPs. This ensured improved cellular uptake and lower toxicity when compared to LNPs formulated without the RGD lipid. Furthermore, codelivery of Cas9 mRNA and sgRNA for *in vitro* gene editing was possible, showing the successful knock down of green fluorescent protein (GFP) in up to 90% of HepG2 cells.^49^ Apart from being used to address cancer, peptides can also have other therapeutic applications. Thus, it was investigated whether LNPs can be used to treat inherited retinal degeneration. Therefore, LNPs must transfect the photoreceptors (PRs), which requires overcoming ocular barriers. To accomplish this, LNPs were functionalized with peptides. Based on an M13 bacteriophage-based heptameric peptide phage display library, the most promising peptides were identified. The peptides were connected after formulating the LNPs via a DSPE-PEG2000-maleimide or DSPE-PEG2000-carboxy-NHS linker which was incorporated at up to 0.3% of the total lipid content. Thus, it was possible to give LNPs the ability to transfect PRs in nonhuman primates, with the peptide sequence SPALHFL.^57^

A further example shows how CPP-decorated LNPs were used to achieve efficient internalization into B16F10 murine melanoma. To achieve this, the lipid DOPE was functionalized with a CPP derived from protamine (RRRRRRGGRRRRG) to form the lipid peptide conjugate (DOPE-CPP). Interestingly, DOPE-CPP (6 mol % to total lipids of LNP) was added postformulation to incubate the LNPs for 30 min at 40 °C. Impressively, the presence of DOPE-CPP increased the stability of the LNPs containing fluorescence-labeled siRNA. The CPP-LNPs were efficiently internalized into B16F10 murine melanoma cells, while LNP without CPP was hardly internalized into these cells.^58^

Research is also continuing on new CPPs for liposomes and LNPs. For example, Sagimoto et al. investigated the influence of a new lipid functionalized with the peptide sequence KK-(EK)4 with regard to cellular uptake. Lipid-based formulations are formulated by mixing the dissolved lipids and subsequently removing the solvent. In this specific case 1% of the composition was the KK-(EK)4-lipid. The liposomes as a reference were treated with 6 mol % at 60 °C for 1 h postformulating. For both liposomes and LNPs a 2–3 fold increase in cytoplasmic fluorescence signal in A549 cells could be observed, in the case for the liposomes referred to a rhodamine dye and for the LNPs due to protein expression levels of the luciferase protein. In both cases the samples were compared to the nonfunctionalized particles and explained by the increased endosomal escape efficiency.^42^

However, none of these examples have completely eliminated off-targeting or dramatically increased the efficiency of delivery, which represent two major challenges for future nanomedicine.

## 6 Lipid-Based Nanoparticles Functionalized with Fusogenic Peptides as CPPs for *In Vitro* and *In Vivo* Drug Delivery

Receptor-targeted nanoparticles are usually internalized via the endolysosomal pathway, which often leads to degradation of the payload in the lysosomes.^59^ Therefore the transport efficiency into the cytoplasm is limited for particles due to poor endosomal escape. Thus, other strategies to deliver cargo to cells are required. Besides receptor targeting, peptides can mediate the cellular uptake of functionalized lipid-based nanoparticles via a biomimetic mechanism using a coiled-coil interaction. Examples showed that they can be easily inserted into cells, liposomes, vesicles, and lipid nanoparticles (LNPs).^25,29,30^ By modifying cells and vehicles, it could be shown that the uptake was drastically increased and selectivity over modified and nonmodified cells was achieved.

### 6.1 Enhanced Liposomal Drug Delivery *In Vitro* and *In Vivo* Using Fusogenic Coiled-Coil-Forming Peptides

Originally, a coiled-coil peptide system was designed by using peptides E and K with three heptad repeats conjugated to cholesterol with a PEG_4_ linker, yielding lipopeptides cholesterol-PEG_4_-E3 and -K3 (CPE3 and CPK3). When pretreating cells with CPK3 and functionalizing liposomes with CPE3, cell membrane docking was observed, but no membrane fusion occurred.^60^ Subsequently, the number of heptad repeats was increased to four, yielding CPE4 and CPK4. Using these optimized lipopeptides, the delivery system achieved the intracellular delivery of a fluorescent dye: HeLa cells pretreated with CPK4 showed the efficient uptake of CPE4-functionalized liposomes and subsequent fluorescent payload delivery, whereas controls showed no or much lower intracellular fluorescence.^1^

Later, it was demonstrated that cell pretreatment with CPK4 could be replaced by genetically modifying cells, introducing a stable expression of peptide K4 fused to a transmembrane domain. The delivery mechanism was also verified *in vivo* by xenografting the genetically modified HeLa-K cell line in zebrafish embryos. Upon intravenous injection, CPE4-functionalized liposomes delivered their fluorescent payload to HeLa-K cells, whereas in control experiments in the absence of one of the peptides, no uptake was observed.

Subsequently, it was demonstrated that this system could enhance the delivery of liposome-encapsulated FDA-approved anticancer agent doxorubicin: the cell viability of HeLa-K cells treated with CPE_4_-functionalized liposomal doxorubicin was reduced by 80%, whereas controls showed a negligible reduction in cell viability. Moreover, *in vivo* in xenografted zebrafish embryos, a 5-fold lower concentration of this formulation achieved a greater anticancer effect on HeLa-K cells than nonfunctionalized liposomal doxorubicin or free doxorubicin at the dose used in the clinic.^2^

**Figure 3:**
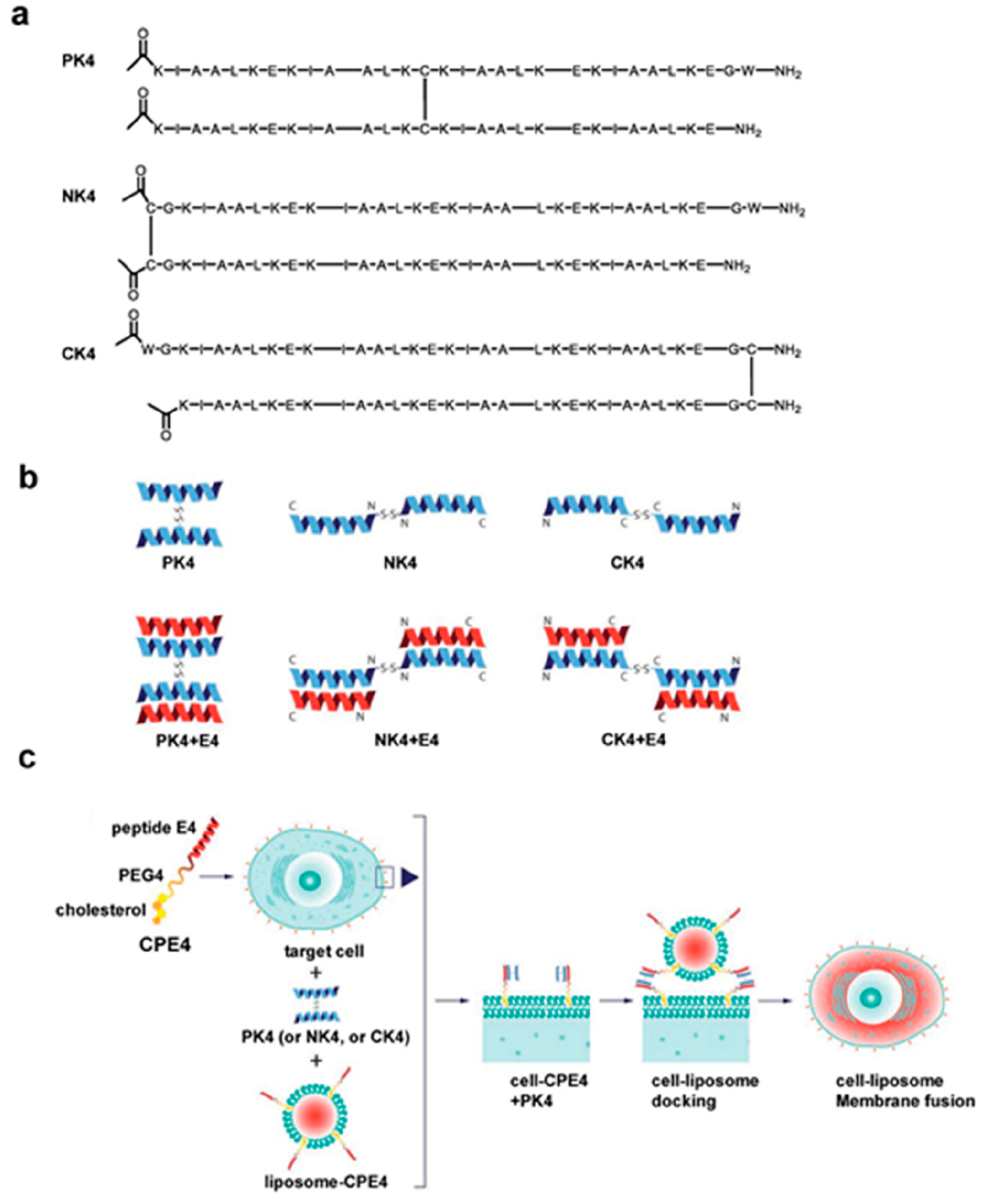
Schematic illustration of the cell–liposome membrane fusion process triggered by K4 dimers and E4. a) Peptide sequence information of K4 dimers. b) Schematic representation of K4 dimers and coiled-coil structures of K4 dimers with complementary E4 peptides. c) Liposomal drug delivery to cells through membrane fusion induced by different coiled-coil peptides. Reproduced with permission from ref (3). Copyright Wiley 2023.

Several years later, it was rationalized that dimers of peptide K might enhance liposomal drug delivery even further through their affinity for both fluid phospholipid membranes and peptide E. Upon membrane binding, peptide K induces positive membrane curvature and destabilization, facilitating membrane fusion.^61^ Thus, through simultaneous competing interactions with both peptide E and the membrane, peptide K could enhance membrane fusion. A stacked dimer conformation, named PK4, allowing higher-order self-assembly, showed coiled-coil formation with peptide E4 at a 1:2 stoichiometric ratio (Figure 3a,b). Moreover, this dimer conformation showed much stronger interaction and subsequent fusion events with fluid phospholipid membranes than did linear dimer versions (NK4 and CK4) in lipid- and content-mixing assays. The membrane affinity of PK4 was also found to be higher than that of the K4 monomer. Furthermore, cholesterol-PEG_4_-K4 (CPK4) conjugates were used to trigger membrane fusion; however, the lipid mixing efficiency upon PK4/CPE4 interaction was found to be higher than that of CPK4/CPE4.

*In vitro*, the different K4-dimer versions were used to label HeLa cell membranes (Figure 3c). Similarly, CPE4/PK4-pretreated cells showed the strongest membrane labeling upon binding with fluorescent E4 or interaction with CPE4-functionalized liposomes. Moreover, enhanced binding also translated into increased delivery of liposomal cargo, as demonstrated with the delivery of fluorescent DNA-binding dye PI, with intracellular fluorescence intensity showing the same trend as in the membrane labeling experiment.

Finally, it was demonstrated using doxorubicin as CPE4-liposomal cargo that the cell viability of CPE4/PK4-pretreated HeLa cells was more potently reduced compared to when the other K4-dimers or the monomer was used. Importantly, endocytosis inhibitor assays confirmed that the predominant doxorubicin delivery route was via direct coiled-coil-induced membrane fusion rather than endocytosis.^3^

### 6.2 Lipid Nanoparticle Functionalization with Coiled-Coil Peptides for Enhanced mRNA Delivery *In Vitro*

Similar to liposomes, LNPs are primarily internalized through endolysosomal pathways, and it has been demonstrated that a sheer <5% of internalized RNA in LNPs escapes the endosomes and enters the cytosol to allow its therapeutic effect.^62^ Thus, other delivery routes for LNP-based nucleic acid delivery, such as via membrane fusion, might provide solutions to this limited endosomal escape capability.

**Figure 4:**
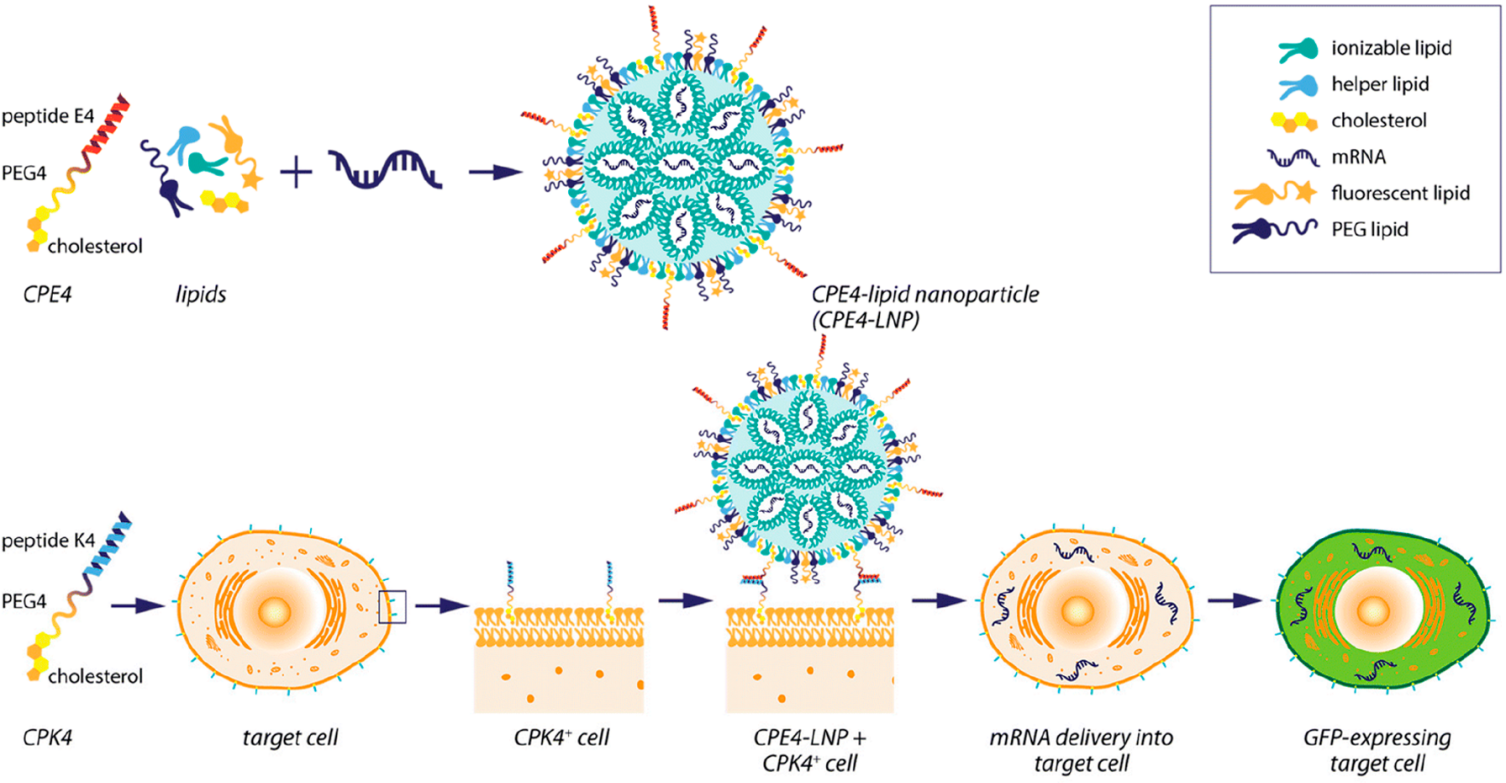
Schematic representation of the nonviral lipid nanoparticles (LNPs) that induce efficient mRNA delivery within cells when modified with fusogenic coiled-coil peptides. Reproduced from ref (32) with permission from the Royal Society of Chemistry.

Recently, the success of liposomal internalization through membrane fusion induced by coiled-coil-forming lipopeptides CPE and CPK was extended to LNPs (Figure 4). ONPATTRO-like LNPs encapsulating EGFP-mRNA were functionalized with 1 mol % CPE3 or CPE4 (differing in one heptad repeat) and were added to HeLa cells pretreated with CPKn. In line with previous liposome results, the CPK4/CPE4 pair showed enhanced cellular uptake compared to that of the CPK3/CPE3 pair. An analysis of the physicochemical properties of LNP formulations with and without CPE4-functionalization showed no major changes.

The cellular uptake of CPE4-LNPs encapsulating AF488-labeled nucleic acids was studied in detail using confocal imaging and flow cytometry. Colocalization of lipid-dye conjugate DOPE-LR and AF488-nucleic acid decreased as the dye remained plasma membrane-bound, whereas the AF488 signal was localized to the cytoplasm. This indicates membrane fusion as the internalization route of these LNPs, whereas controls lacking one of the lipopeptides showed a much lower intracellular AF488 signal. Flow cytometry revealed fast uptake kinetics for coiled-coil-induced LNP uptake, as only 15 min post-treatment 99.9% of cells were AF488-positive. In addition, the mean fluorescence intensity (MFI) was much higher compared with that of control groups lacking one or both lipopeptides. Similar results were obtained when using Chinese hamster ovary (CHO) cells, murine fibroblasts NIH/3T3, and generally hard-to-transfect Jurkat T cells. Endocytosis inhibitor assays revealed that indeed, in line with previous liposome results, coiled-coil-induced membrane fusion was the predominant internalization route of CPE4-LNPs, whereas unfunctionalized LNPs mainly entered cells through clathrin-mediated endocytosis. Thus, the endolysosomal pathway was avoided, resulting in enhanced nucleic acid delivery.

Subsequently, EGFP expression in HeLa cells was compared for CPE4-LNPs, unfunctionalized LNPs, and the commercial transfection reagent Lipofectamine 3000. Flow cytometry revealed large differences in EGFP-positive CPK4-pretreated cells between CPE4-LNPs (99.9%), Lipofectamine 3000 (54.7%), and unfunctionalized LNPs transfecting HeLa without CPK4-pretreatment (20.9%). Moreover, the EGFP MFI was 50-fold higher for CPK4-cells treated with CPE4-LNPs compared to that of the control without lipopeptide. Similar results were obtained for cell lines CHO and NIH/3T3, whereas the hard-to-transfect Jurkat T cells showed over 3-fold higher EGFP expression compared to the control without lipopeptide. Finally, a cytotoxicity assay revealed no significant differences in the cytotoxicity between CPE4- and unfunctionalized LNPs. Altogether, these results showed that poor endosomal escape can be avoided by LNP internalization through CPE4/CPK4-induced membrane fusion, resulting in high transfection efficiency and enhanced mRNA expression *in vitro*.^32^

### 6.3 CPE4/CPK4 Coiled-Coil Peptides for Enhanced Local LNP-Mediated mRNA Delivery *In Vivo*

Building on this *in vitro* success, the CPE4/CPK4 system was applied *in vivo* to improve local mRNA-LNP transfection. To streamline the target cell pretreatment method, a 1-step incubation protocol compatible with *in vivo* applications was developed. This method comprised the premixing of micellar CPK4 and CPE4-modified LNPs before addition to target cells or local administration *in vivo*. To ensure no major changes in the physicochemical properties of LNPs upon mixing, size measurements by dynamic light scattering (DLS) were performed after mixing in PBS or 10% fetal calf serum (FCS). Only a minor size increase but no aggregation was observed upon mixing of CPE4-LNPs and micellar CPK4. In addition, cryo-TEM confirmed that the cores of CPE4-LNPs showed the same morphology before and after mixing.

**Figure 5:**
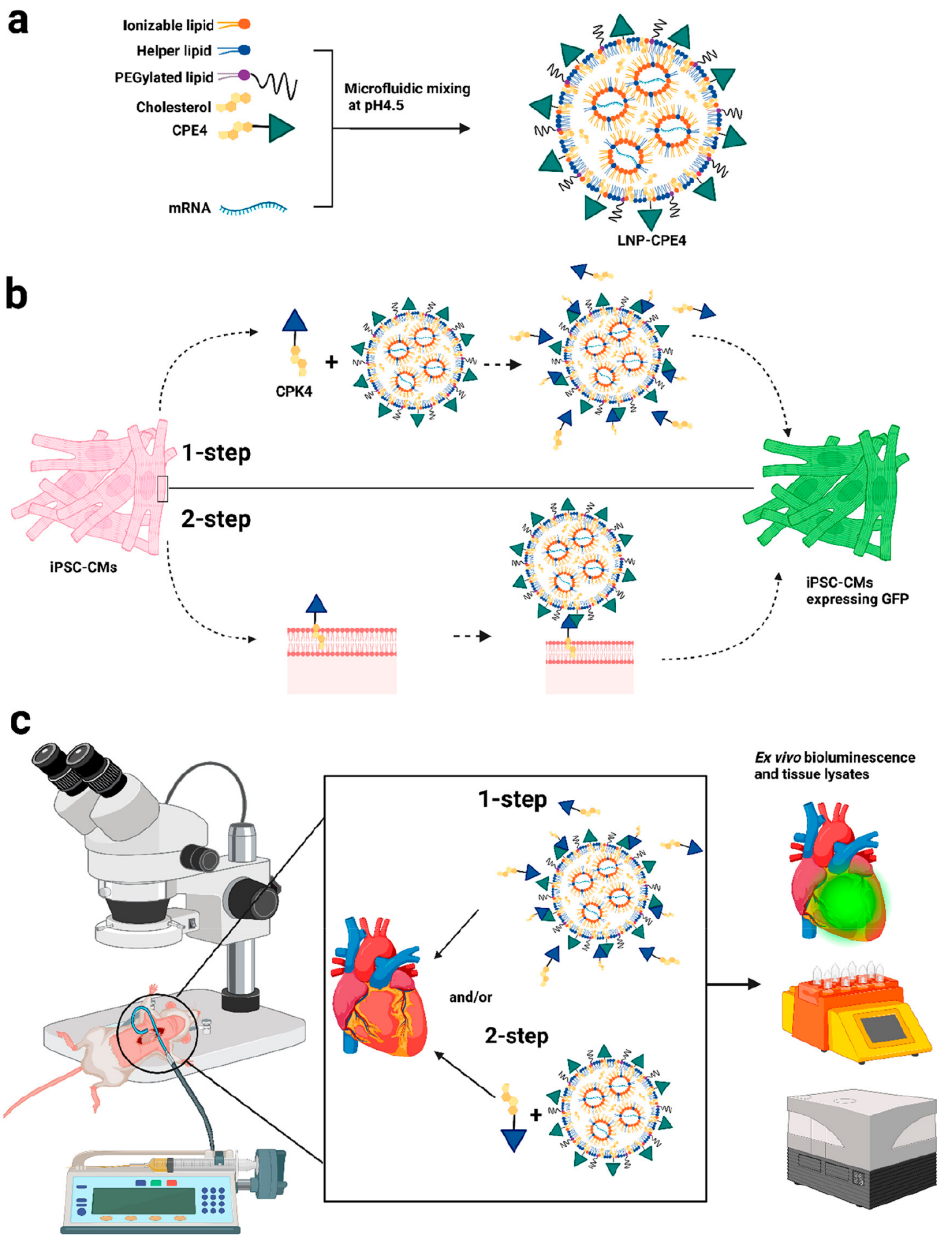
Overview of the LNP formulation and delivery *in vitro* and *in vivo*. (a) Schematic illustration of mRNA encapsulating LNP-CPE4. (b) Fusogenic coiled-coil peptide-modified lipid nanoparticles (LNPs) for EGFP-mRNA delivery in iPSC-CMs. In the 1-step protocol, CPK4 and LNP-CPE4 are premixed and added to the cells. In the 2-step protocol, cells were first pretreated with CPK4 before incubation with LNP-CPE4. (c) Schematic illustration of the intramyocardial administration of LNPs encapsulating luciferase-mRNA. Reproduced with permission from ref (4). Copyright ACS 2023.

Subsequently, the method was verified *in vitro* using HeLa cells by transfection according to the original 2-step method in which cells were pretreated with CPK4, followed by incubation with CPE4-LNPs encapsulating EGFP-mRNA, or according to the 1-step protocol in which cells were incubated with a premixture of CPE4-LNPs and micellar CPK4 (Figure 5). Flow cytometry revealed that out of the different CPK4/CPE4 ratios tested, an equimolar ratio performs the best and resulted in roughly 4-fold-higher EGFP expression compared to the original 2-step method. Next, the 1-step protocol was also verified using induced pluripotent stem cell-derived cardiomyocytes (iPSC-MCs). iPSC-MCs are the most promising cells for cardiac repair due to their indefinite proliferation and ability to differentiate into different cardiac lineages such as smooth muscle cells, endothelial cells, and cardiac progenitors.^63^ Consistent with the results obtained for HeLa cells, the 1-step protocol yielded a 19-fold-higher EGFP expression compared to the control without lipopeptide and roughly doubled the expression compared to commercial mRNA transfection reagent Lipofectamine MessengerMAX.

Subsequently, the system was tested *in vivo* in mice by intramyocardial administration. A pilot study to assess the efficacy and biodistribution of unmodified luciferase mRNA-LNPs administered intravenously showed expression mainly in the liver, with little to no expression in the heart. Modification of the LNPs with coiled-coil peptides did not result in a significantly different biodistribution profile. Therefore, local administration into the heart might be crucial for effective cardiac regenerative therapy after myocardial infarction. Although mRNA expression in the liver followed by the spleen remained higher, expression in the heart was significantly enhanced for LNPs modified with coiled-coil peptides compared to the controls. It was postulated that selectivity for the heart might be higher in larger mammals since intramyocardial injection in live mice is technically challenging and results in significant direct flush-out into the bloodstream. Furthermore, the safety profile of lipopeptide-modified LNPs was tested by measuring serum levels of liver enzymes 24 h after administration. No significant deviations from the controls were observed for the coiled-coil-modified LNPs.

To assess whether the system performed similarly for a broad range of LNPs, two clinically approved ionizable lipids, ALC-0315 (Pfizer/BioNTech) and SM-102 (Moderna), were used to formulate LNPs modified with coiled-coil peptides, and the transfection and flow cytometry experiments were repeated for HeLa, Jurkat T cells, and iPSC-MCs according to the 1-step approach. CPK4/CPE4-functionalized LNP formulations consistently showed enhanced mRNA transfection compared to that of their unfunctionalized counterparts in these cell lines. Altogether, functionalizing mRNA-LNPs with fusogenic coiled-coil peptides using a 1-step mixing approach significantly enhances mRNA transfection in HeLa and hard-to-transfect iPSC-CMs and Jurkat cells *in vitro* and improves local mRNA transfection upon intramyocardial administration *in vivo*.^4^

## 7 Conclusions

Liposomes and LNPs are highly flexible to desired applications and have already demonstrated their relevance and enormous potential for future healthcare. The therapeutic effect depends mainly on the interior of the particles, while the fate (biodistribution and targeting properties) relies on the surface constitution. It has been shown that for surface functionalization, peptides are perfectly suited. However, surface functionalization of such nanoparticles is not trivial and strongly depends on the nanoparticles and peptides used. Since a custom peptide is required for each application and this cannot simply be transferred from one nanoparticle to another, extensive modifications and screening are required. To avoid such time-consuming and costly procedures, complementary fusogenic coiled-coil peptides can be used, which have shown promising results in diseases that allow for local injections. Thus, the tissue to be addressed is labeled by a local injection with a peptide, which allows increased specific cellular uptake of particles with the complementary peptide.

Since local injections are not an option for every disease, other alternatives need to be developed. One way to achieve clinical applications could be the injection of a PEGylated CPK that can be locally unshielded by irradiation with light or the conjugation of the K4 peptide with cancer-specific cell membrane antibodies.

Additionally, increased cytosolic delivery is achieved by avoiding the endolysosomal pathway. Undoubtedly, this approach is promising for localized disease patterns and should continue to be explored in future studies.
